# Mortality Associations With DNA Methylation-Based Biological Aging and Physical Functioning Measures Across a 20-Year Follow-up Period

**DOI:** 10.1093/gerona/glad026

**Published:** 2023-01-22

**Authors:** Tiina Föhr, Katja Waller, Anne Viljanen, Taina Rantanen, Jaakko Kaprio, Miina Ollikainen, Elina Sillanpää

**Affiliations:** Faculty of Sport and Health Sciences, Gerontology Research Center (GEREC), University of Jyväskylä, Jyväskylä, Finland; Faculty of Sport and Health Sciences, University of Jyväskylä, Jyväskylä, Finland; Faculty of Sport and Health Sciences, Gerontology Research Center (GEREC), University of Jyväskylä, Jyväskylä, Finland; Faculty of Sport and Health Sciences, Gerontology Research Center (GEREC), University of Jyväskylä, Jyväskylä, Finland; Institute for Molecular Medicine Finland (FIMM), University of Helsinki, Helsinki, Finland; Institute for Molecular Medicine Finland (FIMM), University of Helsinki, Helsinki, Finland; Minerva Foundation Institute for Medical Research, Helsinki, Finland; Faculty of Sport and Health Sciences, Gerontology Research Center (GEREC), University of Jyväskylä, Jyväskylä, Finland; Institute for Molecular Medicine Finland (FIMM), University of Helsinki, Helsinki, Finland; Central Finland Wellbeing Services County, Jyväskylä, Finland

**Keywords:** Epigenetic clock, Timed Up and Go test, Twins, Walking speed

## Abstract

**Background:**

Measures of biological aging range from DNA methylation (DNAm)-based estimates to measures of physical abilities. The purpose of this study was to compare DNAm- and physical functioning-based measures of biological aging in predicting mortality.

**Methods:**

We studied 63- to 76-year-old women (*N* = 395) from the Finnish Twin Study on Aging (FITSA). Participants’ biological age (epigenetic clocks DNAm GrimAge and DunedinPACE) was estimated using blood DNAm data. Tests of physical functioning conducted under standardized laboratory conditions included the Timed Up and Go (TUG) test and 10-m walk test. Mortality hazard ratios were calculated per every 1 standard deviation (*SD*) increase in the predictor. Cox regression models were conducted for individuals and twin pairs, the latter controlling for underlying genetic effects. The models were adjusted for known lifestyle predictors of mortality.

**Results:**

During the follow-up period (mean 17.0 years, range 0.2–20.3), 187 participants died. In both the individual-based and pairwise analyses, GrimAge and both functional biomarkers of aging were associated with mortality independent of family relatedness, chronological age, physical activity, body mass index, smoking, education, or chronic diseases. In a model including both the DNAm-based measures and functional biomarkers of aging, GrimAge and TUG remained predictive.

**Conclusions:**

The findings suggest that DNAm GrimAge and the TUG test are strong predictors of mortality independent of each others and genetic influences. DNAm-based measures and functional tests capture different aspects of the aging process and thus complement each other as measures of biological aging in predicting mortality.

## Background

While chronological age is a major risk factor for functional impairments, chronic diseases, and mortality, there is a great degree of heterogeneity among older individuals in terms of their health and physical functioning (1). Thus, chronological age is not a sufficient marker for understanding and measuring healthy aging, and the search for additional reliable markers of biological age has been ongoing for decades (2). Biological aging includes physiological processes at the molecular, cellular, organ, and system levels, and it may predict those at the greatest risk of age-related diseases and disabilities as well as premature mortality. Measures of biological aging range from DNA-based estimates to measures of physical abilities (3–6).

Epigenetic clocks are based on changes in the DNA methylation (DNAm attachment of a methyl group to C-5 of cytosine bases in the context of cytosine–phosphate–guanine [CpG] dinucleotide in a DNA strand) levels over time. Epigenetic mechanisms—of which DNAm is the most studied—regulate gene expression and help us adapt to different environments and lifestyles. Thus, epigenetics forms a fundamental link between genetics and environment/lifestyle. The epigenetic clock DNAm GrimAge, a combination of DNAm-based surrogate biomarkers for health-related plasma proteins and smoking pack-years as well as sex and chronological age, was developed to predict mortality (7), and has been shown to outperform previous clocks in this regard (8,9). It is associated with the key hallmarks of aging, such as mitochondrial dysfunction and cellular senescence (10,11). A next-generation DNAm-based biomarker of the pace of aging, DunedinPACE (Pace of Aging Calculated from the Epigenome), is associated with mortality and may add incremental prediction beyond that of GrimAge (12,13).

Measures of physical functioning mirror the physiological changes that occur with aging (14). On the other hand, if DNAm-based measures of aging require blood chemistries and access to genotyping, functional measurements are inexpensive and easy to accomplish, as they require little more than a stopwatch in terms of equipment. For instance, short-distance walking tests and composite measures of physical functioning, such as the Timed Up and Go (TUG) test, can be performed in a limited space without special equipment, thus making them simple and safe to accomplish. The TUG test includes transfer tasks (standing up and sitting down), walking, and turning, incorporating neuromuscular components such as balance, power, and agility (15). Both walking speed (16–18) and the TUG test (17–23) have been shown to predict the risk of mortality.

Biological aging process is a complex phenomenon shaped by genetic inheritance as well as environmental and lifestyle factors. Twin study designs are ideal for examining the associations between complex traits, where multiple factors influence the exposure (e.g., biological aging) and outcome (e.g., mortality) being studied. Twin pairs share the same sex and age, all (monozygotic [MZ] pairs) or half (dizygotic [DZ] pairs) of their segregating genetic polymorphisms, and mostly the same intrauterine and childhood environment, thus allowing for the better control of lifestyle and genetic factors in studies examining the association of epigenetic aging and physical functioning with mortality. The purpose of this study was to investigate the association of 2 DNAm-based measures—epigenetic clock GrimAge and DunedinPACE—and physical functioning-based measures—the TUG and 10-m walk tests—with mortality while acknowledging the effect of education and several lifestyle factors. Based on the current literature, we hypothesized that all these measures predict mortality. However, to the best of our knowledge, no previous study has investigated these specific predictors of mortality within the same cohort. Therefore, little is known about how these 2 sets of physiological aging measures interact as well as whether they are associated with mortality independently of each other.

## Method

### Participants and Study Design

The female participants in the present study participated in the Finnish Twin Study on Aging (FITSA). The participants in the FITSA were recruited from the Older Finnish Twin Cohort, which is comprised of all same-sex twin pairs born before 1958 with both co-twins alive in 1975 (24). The FITSA was set up to investigate genetic and environmental effects on the disablement process in older female twins. An invitation to participate in the FITSA was sent to 414 female twin pairs. The final sample included 114 DZ and 103 MZ twin pairs (434 individuals) aged 63–76 years. The participants were informed about the study and signed a written consent form prior to the laboratory examinations, which took place from 2000 to 2001. The recruitment process for the FITSA has been described in detail previously (25,26). Participants with available DNAm and physical functioning (TUG and 10-m walk) data were included in the present study (*N* = 395).

### Biological Aging

#### DNAm age

In our previous paper, we described the generation, preprocessing, and normalization of the DNAm data (27). Briefly, we determined genome-wide DNAm from blood samples using an Illumina EPIC BeadChip, and the data were preprocessed with the R package *minfi*. Detection *p* values comparing the total signal for each probe to the background signal level were calculated to evaluate the quality of the samples (28). Further analysis excluded samples of poor quality (mean detection *p* > .01). To normalize the data, a single-sample Noob normalization method was used (29).

The epigenetic age estimate, DNAm GrimAge, was calculated using an online calculator (https://dnamage.genetics.ucla.edu/new). For this measure, in the analysis, we used the age acceleration measure, which describes the difference between chronological age and epigenetic age estimates. Age acceleration was defined as the residual from regressing the estimated epigenetic age on the chronological age. The DunedinPACE estimator gives an estimate for the pace of biological aging in years per calendar year (12,13). It was calculated using a publicly available R package (https://github.com/danbelsky/DunedinPACE).

#### Physical functioning

The participants took part in baseline laboratory measurements. Before the measurements of physical functioning, participants’ health status, chronic conditions, and medications were carefully evaluated by a physician during a 30-minute clinical examination. Standardized laboratory measurements of physical functioning, including the TUG test and 10-m walk test, were conducted by trained staff, most of whom were physiotherapists. The participants wore walking shoes or sneakers, and the use of any walking aids they would normally require was allowed in the tests. Both tests were done twice, with the faster performance (in seconds) selected for the analyses.

The TUG test was used to assess the participants’ mobility, balance, and walking ability (30). The participants were verbally and visually instructed to rise from a chair, walk 3 m, turn around, walk back to the chair, and sit down.

In the 10-m walk test with a flying start, 3 m was allowed for acceleration, and participants’ walking time (in seconds) over 10 m was recorded in the laboratory corridor using photocells for timing (26). The participants were instructed to walk as fast as possible without compromising their safety.

### Covariates

Information regarding the known predictors of mortality (e.g., length of education, cigarette smoking, alcohol consumption, physical activity, body mass index [BMI], and chronic diseases) was obtained from the participants’ interviews, questionnaire data, and anthropometric measurements at baseline. The length of education (years) was self-reported. Smoking and alcohol consumption were assessed via a standardized questionnaire. Based on the participants’ responses to a detailed smoking behavior and history questionnaire, smoking status (current, former, or never) was determined, and the lifelong history of exposure to smoking was calculated in pack-years (equivalent to smoking 1 pack [20 cigarettes] per day for a year). Alcohol use was measured with beverage type-specific items in regard to frequency and quantity and converted into the number of grams of absolute ethanol per day. For descriptive purposes, participants were further categorized as abstainers, light drinkers (3 or fewer drinks per week), moderate drinkers (more than 3 but no more than 7 drinks per week), and heavy drinkers (on average, more than 1 drink per day). With slight modifications, the scale developed by Grimby (31) was used as a measure of self-reported physical activity. For descriptive purposes, the participants were further divided into 3 groups accordingly: mainly sedentary (groups 0–1), light physical activity (group 2), and moderate to vigorous physical activity (groups 3–6). BMI values were determined based on weight and height (weight in kilograms divided by the square of height in meters) and measured by trained research staff. Chronic diseases were first self-reported and then confirmed during a medical examination conducted by a physician. The chronic diseases considered here included chronic cardiovascular, pulmonary, neurological, musculoskeletal diseases, type 2 diabetes, and all cancers. These are reported more precisely in our previous papers (9,32).

### Mortality Follow-up and Statistical Analyses

The mortality follow-up began on the date the participant participated in the laboratory measurements and the blood sampling for the genome-wide DNAm analysis was conducted (from 2000 to 2001). Follow-up continued through December 31, 2020, with the all-cause mortality analyzed during this period. The all-cause mortality data—with exact death dates, causes of death, and emigration from Finland status—were available from Statistics Finland.

### Individual-Based Analyses

First, using a Cox proportional hazard model adjusted for family relatedness and age, we conducted an individual-based (i.e., twins within a pair and single co-twins were studied as individuals) mortality analysis. We calculated hazard ratios (HRs) for 1 standard deviation (*SD*) increase in GrimAge, DunedinPACE, TUG test, and 10-m walk test with their 95% confidence intervals (95% CI) for the 395 study participants (Model 1). Further, we adjusted Model 1 for education years, smoking (i.e., smoking status and pack-years), BMI, and physical activity by adding 1 covariate at a time to the model. We also carried out the analyses with multivariable adjustments. The full model (Model 1 + lifestyle factors) included adjustments for family relatedness, age, smoking, BMI, physical activity, and alcohol consumption. Following this, we included an adjustment for education years on top of the lifestyle factor-adjusted Model 1 (Model 2). Finally, we included adjustments for the presence of selected chronic diseases in Model 2. To further assess the association, survival curves were plotted according to the age acceleration/physical functioning tertiles of all 4 predictors, and all-cause mortality was investigated by calculating the HRs during follow-up based on these tertiles. Finally, to investigate whether GrimAge, DunedinPACE, the TUG test, and the 10-m walk test contributed to mortality risk independently of each other, we included all the measures in the same model. For a valid comparison of the selected predictors in terms of the strength of their association with mortality, we used a standardized approach to show the per-*SD* increment of mortality HR among the same number and groups of participants.

### Pairwise Analyses

Using the “strata” option for the Stata procedure stcox (StataIC16, StataCorp, Inc., College Station, TX), pairwise analyses were performed with the same models. These analyses were restricted to twin pairs with nonmissing data for both twins. This compares the hazards within pairs rather than to the overall reference category as in standard Cox regression models. Models were generated for all twin pairs and separately for the MZ pairs with an identical genomic sequence and DZ pairs sharing half of their segregating genes. For all the predictors, the effect of zygosity was tested using an interaction term between the predictor and zygosity and then comparing the fit between the models with and without the interaction term.

## Results

### Individual-Based Analysis

Of the 395 individuals in this study, 187 died (47.3%) during the mean follow-up time of 17.1 years (range 0.2–20.3). The baseline characteristics of the participants are presented for all participants as well as according to vital status (alive/dead) at the end of the follow-up (Table 1). Those who died were older as determined by chronological age (mean = 69.6, *SD* = 3.4 vs mean = 67.6, *SD* = 3.2 years), DNAm-based age (GrimAge mean = 61.2, *SD* = 4.4 vs mean = 58.4, *SD* = 3.8 years), and age acceleration (GrimAge mean = 0.51, *SD* = 3.40 vs mean = −0.64, *SD* = 2.86, DunedinPACE mean = 0.99, *SD* = 0.11 vs mean = 0.95, *SD* = 0.11). Additionally, they performed worse on the tests of physical functioning (TUG mean = 9.9, *SD* = 2.2 vs mean = 8.7, *SD* = 1.5 seconds, 10-m walk mean = 6.4, *SD* = 1.5 vs mean = 5.7, *SD* = 1.0 seconds). Furthermore, those who died had lower levels of education (in years) and physical activity as well as more chronic diseases (cardiovascular and type 2 diabetes) compared to those who were alive at the end of follow-up. With regard to smoking, BMI, and alcohol consumption at baseline, no significant differences were found according to the subsequent vital status of the participants.

**Table 1.** Baseline Characteristics of the Female Participants From the Finnish Twin Study on Aging (*N* = 395, age range 63–76 years) Overall and by Vital Status Over a 20-Year Follow-up Period

		Vital Status at the End of the Follow-up		*p* ^a^
Characteristic	All (*N* = 395)	Alive (*N* = 208)	Dead (*N* = 187)	
Age at baseline, y	68.5 (3.4)	67.6 (3.2)	69.6 (3.4)	<.001
DNAm age				
DNAm GrimAge, y	59.7 (4.4)	58.4 (3.8)	61.2 (4.4)	<.001
GrimAge age acceleration	−0.09 (3.18)	−0.64 (2.86)	0.51 (3.40)	.001
DunedinPACE	0.97 (0.11)	0.95 (0.11)	0.99 (0.11)	.001
Timed Up and Go test (s)	9.3 (1.9)	8.7 (1.5)	9.9 (2.2)	<.001
10-m walk test (s)	6.0 (1.3)	5.7 (1.0)	6.4 (1.5)	<.001
Education, y	8.7 (3.1)	9.1 (3.1)	8.3 (2.9)	.013
Cigarette smoking, *N* (%) of participants				.222
Never smokers	345 (87.6)	187 (90.3)	158 (84.5)	
Former smokers	29 (7.4)	13 (6.3)	16 (8.6)	
Current smokers	20 (5.1)	7 (3.4)	13 (7.0)	
Lifetime smoking pack-years				
Former smokers	10.4 (12.9)	12.2 (16.3)	8.9 (9.6)	.532
Current smokers	25.0 (14.7)	22.6 (12.8)	26.2 (16.0)	.612
Body mass index, kg/m^2^	27.9 (4.7)	28.1 (4.9)	27.6 (4.5)	.349
Physical activity group (0–6)	2.3 (1.3)	2.4 (1.2)	2.1 (1.3)	.029
Physical activity group, *N* (%) of participants				.154
Mainly sedentary	106 (26.8)	48 (23.1)	58 (31.0)	
Light physical activity	132 (33.4)	69 (33.2)	63 (33.7)	
Moderate to vigorous physical activity	157 (39.7)	91 (43.8)	66 (35.3)	
Alcohol consumption, g/d	3.2 (5.8)	3.5 (6.0)	2.9 (5.6)	.382
Alcohol consumption, *N* (%) of participants				.318
Abstainer	131 (33.2)	60 (28.8)	71 (38.2)	
Light drinker	192 (48.7)	108 (51.9)	84 (45.2)	
Moderate drinker	52 (13.2)	29 (13.9)	23 (12.4)	
Heavy drinker	19 (4.8)	11 (5.3)	8 (4.3)	
Selected diseases, *N* (%) of participants				
Cardiovascular	218 (55.2)	104 (50.0)	114 (61.0)	.038
Pulmonary	60 (15.2)	28 (13.5)	32 (17.1)	.305
Neurological	6 (1.5)	1 (0.5)	5 (2.7)	.086
Musculoskeletal	218 (55.2)	152 (73.1)	142 (75.9)	.520
Type 2 diabetes	20 (5.1)	5 (2.4)	15 (8.0)	.029
Cancer	49 (12.4)	23 (11.1)	26 (13.9)	.418

*Note*: DNAm = DNA methylation.

^a^Difference between groups according to vital status (alive/dead). Values are means and standard deviations unless otherwise stated.

GrimAge was found to be correlated with the TUG test (*r* = 0.145) and DunedinPACE (*r* = 0.578). DunedinPACE was found to be correlated with both functional biomarkers of aging (TUG *r* = 0.202; 10-m walk *r* = 0.143). Additionally, the TUG test correlated with the 10-m walk test (*r* = 0.742). There was no interaction between these measures as predictors of mortality (test for interaction GrimAge × TUG *p* = .599, GrimAge × DunedinPACE *p* = .652, DunedinPACE × TUG *p* = .778, DunedinPACE × 10-m walk *p* = .865, TUG × 10-m walk *p* = .133).

**Figure 1.**
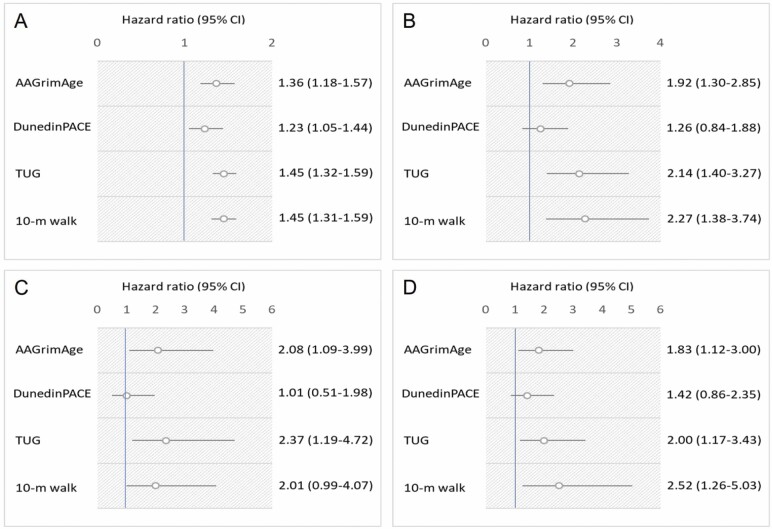
Risks of all-cause mortality per 1-*SD* increase in DNAm GrimAge age acceleration, DunedinPACE, Timed Up and Go test, and 10-m walk test among female participants from the Finnish Twin Study on Aging (*N* = 395, age range = 63–76 years) over a 20-year follow-up period. (A) Individual analyses. (B) Pairwise analyses among all (*N* = 186) twin pairs. (C) Pairwise analyses among monozygotic (*N* = 91) twin pairs. (D) Pairwise analyses among dizygotic (*N* = 95) twin pairs. *Notes:* AAGrimAge = GrimAge age acceleration; CI = confidence interval; DNAm = DNA methylation; *SD* = standard deviation; TUG = Timed Up and Go test.

Both biological aging and functional biomarkers of aging were associated with mortality. In Model 1, including adjustments for family relatedness and chronological age, the mortality HR per every 1-*SD* increase in the predictor variable was 1.36 for GrimAge (95% CI: 1.18–1.57), 1.23 for DunedinPACE (95% CI: 1.05–1.44), 1.45 for the TUG test (95% CI: 1.32–1.59), and 1.45 for the 10-m walk test (95% CI: 1.31–1.59; Figure 1). In Model 2, including adjustments for family relatedness, age, education, all studied lifestyle factors, and chronic diseases, these estimates using GrimAge (HR 1.39; 95% CI: 1.11–1.74), the TUG test (HR 1.43; 95% CI: 1.27–1.60), and the 10-m walk test (HR 1.44; 95% CI: 1.26–1.63) were only marginally affected. The corresponding estimate using DunedinPACE was attenuated to 1.15 (95% CI: 0.96–1.37) (Supplementary Table 1). When all predictors used were analyzed simultaneously in the same model, both GrimAge (HR 1.33; 95% CI: 1.12–1.59) and the TUG test (HR 1.28; 95% CI: 1.04–1.57) remained significantly associated with mortality (Table 2).

**Table 2.** Risks of All-Cause Mortality Per 1-*SD* Increase in DNAm GrimAge Age Acceleration, DunedinPACE, Timed Up and Go Test, and 10-m Walk Test

	Individual Analyses^a^ (*N* = 395)	Pairwise Analyses Among Twins		
		All (*N* = 186) Twin Pairs	Monozygotic (*N* = 91) Twin Pairs	Dizygotic (*N* = 95) Twin Pairs
AAGrimAge	**1.33 (1.12–1.59)**	**2.39 (1.40–4.07)**	**2.77 (1.13–6.76)**	**2.14 (1.10–4.19)**
DunedinPACE	1.00 (0.82**–**1.21)	0.66 (0.38**–**1.16)	0.41 (0.15**–**1.07)	0.85 (0.43**–**1.69)
TUG	**1.28 (1.04–1.57)**	**1.81 (1.08–3.04)**	2.16 (0.87**–**5.38)	1.78 (0.90**–**3.52)
10-m walk	1.16 (0.92**–**1.47)	1.52 (0.85**–**2.69)	1.19 (0.43**–**3.31)	1.61 (0.77**–**3.39)

*Notes*: AAGrimAge = GrimAge age acceleration; DNAm = DNA methylation; *SD* = standard deviation; TUG = Timed Up and Go test. Hazard ratios and 95% confidence intervals are presented in the table. Statistically significant values are bolded.

^a^Adjusted for family relatedness and age.

**Figure 2.**
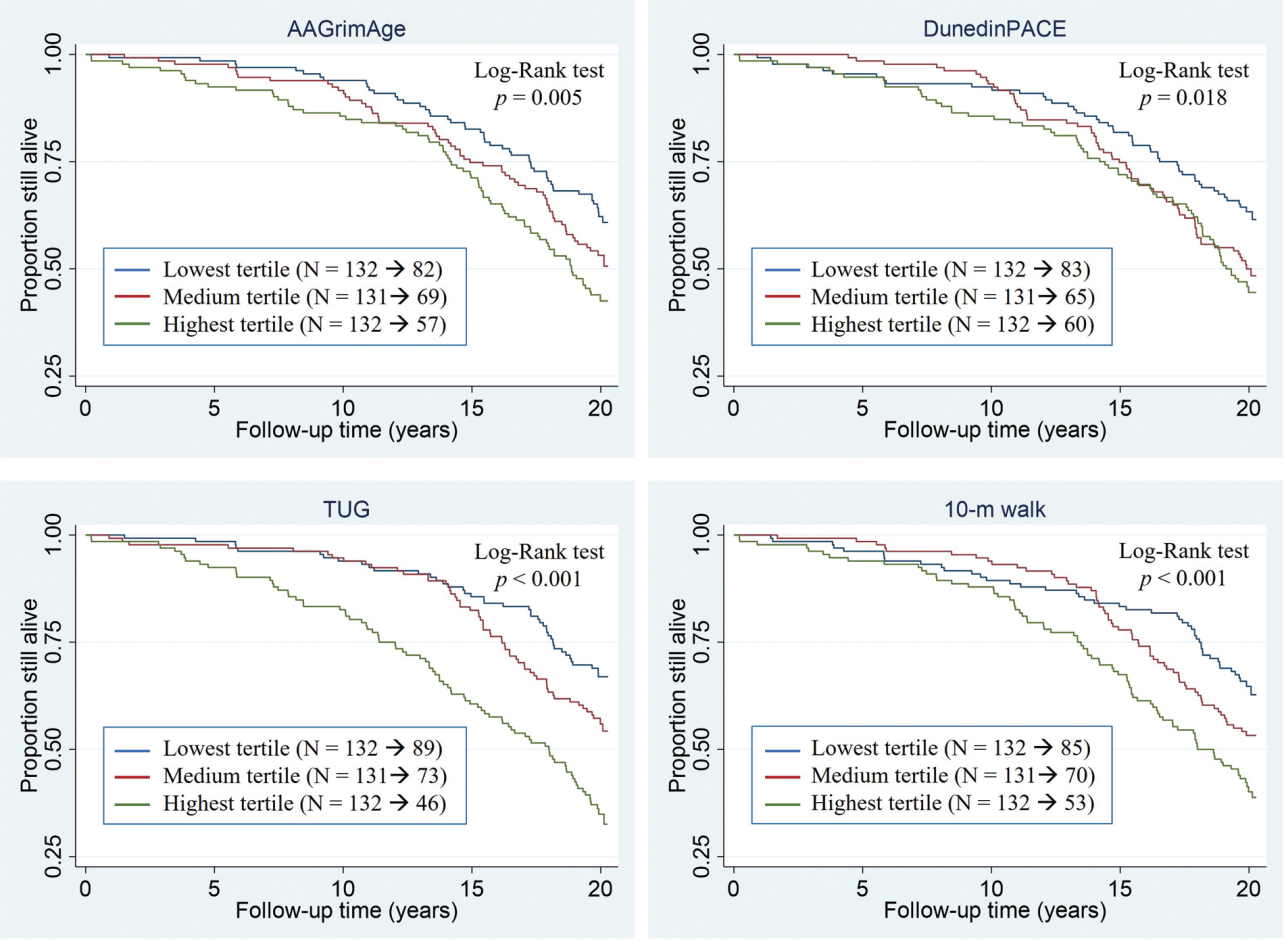
Risks of all-cause mortality per 1-*SD* increase in DNAm GrimAge age acceleration, DunedinPACE, Timed Up and Go test and 10-m walk test according to the tertiles of the predictors. The follow-up period for the female participants from the Finnish Twin Study on Aging (*N* = 395, age range 63–76 years) was 2000–2020. *Notes:* AAGrimAge = GrimAge age acceleration; DNAm = DNA methylation; *SD* = standard deviation; TUG = Timed Up and Go test. For AAGrimAge and DunedinPACE, the highest tertile refers to “fast agers”; for the TUG and 10-m walk tests, it refers to “low physical functioning”.

To further assess the association, survival curves were plotted according to the tertiles of all 4 predictors (Figure 2). Kaplan–Meier survival curves were tested unequal (*p* = .005 for GrimAge, *p* = .018 for DunedinPACE, *p* < .001 for TUG, *p* < .001 for 10-m walk) with a log-rank test. In Model 1, participants in the highest GrimAge tertile (“fast agers”) compared to those in the lowest tertile (“slow agers”) were at a higher risk for mortality (HR 1.68; 95% CI: 1.15–2.46). Likewise, participants in the highest TUG and 10-m walk test tertiles (“low physical functioning”) compared to those in the lowest tertiles (“high physical functioning”) were at a higher risk for mortality (HR 2.48; 95% CI: 1.71–3.59, HR 1.62; 95% CI: 1.10–2.40, respectively). For DunedinPACE, the comparison of the highest tertile with the lowest tertile yielded nonsignificant results (HR 1.29; 95% CI: 0.87–1.91). In Model 2, these estimates using GrimAge (HR 1.51; 95% CI: 1.00–2.26), the TUG test (HR 2.46; 95% CI: 1.65–3.66), and the 10-m walk test (HR 1.68; 95% CI: 1.03–2.75) were only marginally affected. However, in Model 2 including adjustment for chronic diseases, these estimates attenuated and yielded nonsignificant results, except for the TUG test (HR 2.26; 95% CI: 1.50–3.41) (Supplementary Table 2). The variable-specific characteristics and number of deceased participants according to the tertiles of the variable are presented in Supplementary Table 3.

### Pairwise Analysis

To control for genetic and environmental factors shared within a twin pair, pairwise mortality analyses were conducted. Of the 186 twin pairs, at least 1 twin died in 122 pairs during the follow-up period; both twins died in 51 pairs. Among MZ/DZ pairs, at least 1 twin died in 55/67 pairs and both twins died in 24/27 pairs. The within-pair Cox regression, conducted for all twin pairs and separately for the MZ pairs and DZ pairs, accounted for such differences in survival by comparing the co-twins to each other. Except for DunedinPACE, all the measures predicted mortality. In Model 1, the mortality HR per every 1-*SD* increase in the predictor variable was 1.92 (95% CI: 1.30–2.85) for GrimAge, 2.14 (95% CI: 1.40–3.27) for the TUG test, and 2.27 (95% CI: 1.38–3.74) for the 10-m walk test (Figure 1). These estimates were only marginally affected after further adjustments (Supplementary Table 1). Using GrimAge and TUG test, the HRs were systematically but nonsignificantly higher among the MZ pairs in comparison to the DZ pairs (test for interaction in Model 1 GrimAge × zygosity *p* = .982 and TUG × zygosity *p* = .771). The 10-m walk test showed no systematic differences between the MZ and DZ pairs in the pairwise analysis (Supplementary Table 1).

### Sensitivity Analysis

We conducted individual-based sensitivity analyses among the participants with no reported chronic diseases (*N* = 57). In Model 1, the mortality HR per every 1-*SD* increase in the predictor variable was 1.52 for GrimAge (95% CI: 1.19–1.95), 1.34 for DunedinPACE (95% CI: 0.97–1.87), 2.08 for the TUG test (95% CI: 1.06–4.09), and 1.29 for the 10-m walk test (95% CI: 0.69–2.42; data not shown).

## Discussion

There has been consensus that no single measure—or single composite measure—will capture all aspects of the aging process (3,33). In the present study, we investigated 4 potential predictors of mortality, including 2 DNAm-based measures of biological aging and 2 functional biomarkers of aging, among older home-dwelling women. We were able to take into account the effect of genetics, education, and several lifestyle factors. The main findings suggest that physical functioning may be an even stronger predictor of mortality than DNAm-based measures of biological aging. The novelty of this study was its use of the newest epigenetic predictors of biological aging and comparison of how different types of physiological measures differ in their predictive power and how they interact.

We found that DNAm GrimAge, which was developed to predict life span and health span (10,34), and both measures of physical functioning showed robust associations with mortality independent of genetic influences. More specifically, faster aging pace—measured using the GrimAge algorithm—and lower physical functioning were associated with a higher risk of all-cause mortality. The standardized measurements allowed us to compare the predictive abilities of the DNAm-based and functional biomarkers of aging. The TUG test effect sizes were similar to those of the 10-m walk test, and both of these were slightly higher when compared with GrimAge—which showed effect sizes similar to our previous study (9) (which had a shorter follow-up period). DunedinPACE showed the lowest effect size in the individual analysis, with attenuated and nonsignificant HRs in the pairwise analysis. To assess whether these variables predicted mortality independently of each other, they were analyzed simultaneously in the same model. GrimAge, which had the largest effect size, and the TUG test were found to be independent predictors of all-cause mortality.

TUG and 10-m walk tests are easy to conduct, as they are safe to administer, can be performed in a small space, and are cheap tests requiring no special equipment. Previous studies have found both walking speed (16–18) and the TUG test (17–23) to be associated with mortality. Our results are in line with these previous studies (16–23), showing that participants who performed worse in the TUG and 10-m walk tests were at a higher risk for mortality compared to those who accomplished the tests in a shorter amount of time (indicating better physical function). We found that the TUG test was a stronger predictor of mortality compared to the 10-m walk test. In previous research, comfortable walking speed has been found to be a stronger predictor of mortality than the TUG among community-dwelling women (17). However, the present study is not quite comparable to previous research. While, in the present study, participants were asked to accomplish the tests as quickly as possible, other studies have also utilized comfortable/usual walking speed (17,35) and, in the most widely used version of the TUG, participants are asked to complete the task at a comfortable walking speed (15). Additionally, previous studies differ according to their study population and the length of the follow-up period. For instance, some studies have utilized community-dwelling populations while others—such as the present study—have utilized home-dwellers. Additionally, the mortality follow-up period exceeded 10 years in only 3 of the studies (17,19,20). To our knowledge, no previous studies have shown the association between these functional biomarkers of aging and mortality over a follow-up period exceeding 20 years. Additionally, to our knowledge, this is the first study suggesting that the TUG test and walking speed are associated with mortality independent of genetic inheritance and early childhood environmental factors as when we examined the associations within the twin pairs by conducting pairwise analyses, these associations remained significant. However, it is noteworthy that functional biomarkers of aging may be particularly useful, and better than epigenetic clocks, in predicting mortality in older adults. Though, this is less clear for young and middle-aged adults as the changes that occur with aging at the molecular, cellular, and intercellular levels take many years before deteriorating into decreases in physical function (36).

The present study is one of the first to include both DNAm GrimAge and DunedinPACE. The equal test–retest reliability of GrimAge and DunedinPACE has been reported to be higher in comparison to the other epigenetic clocks (12). The next-generation DNAm-based biomarker of the pace of aging, DunedinPACE, has been reported to show effect sizes similar to those of GrimAge in terms of mortality. The results of this study suggest that DunedinPACE, which was developed using DNAm data from 45-year olds and functional data from 26- to 45-year olds, may have limited usability among older populations. The present study found lower effect sizes of DunedinPACE for mortality compared to GrimAge. Further, the attenuated results from the pairwise analysis indicate that the association of DunedinPACE with mortality was partly explained by genetic and early childhood environmental influences. The present study is unique in that it included both DNAm-based measures and functional biomarkers of aging. Thus, we were interested to investigate whether physical functioning reflects the association between DNAm-based biological aging and mortality. Previously, DNAm GrimAge has been found to be associated with decline in physical functioning over a 16-year follow-up period (37). Additionally, our previous study found DNAm GrimAge to be associated with a decline in physical functioning over a 3-year follow-up period among the study group of the present study (32). Also, DunedinPACE has been suggested to be associated with functional decline in midlife adults (12). However, even though GrimAge correlated with the TUG test and DunedinPACE correlated with both the TUG test and 10-m walk test, we did not find an interaction between these measures as predictors of mortality. Thus, the present findings suggest that DNAm-based biological aging is not related to the association between physical function and mortality.

Several limitations of this study need to be considered. First, the present study included only women, which may limit the generalizability of the results. However, previous studies on TUG test–mortality associations that have included both men and women have not reported gender differences. For example, during a mean 11.8-year follow-up period, Bergland et al. (20) found that the association between the TUG test and all-cause mortality was equally strong among male and female home-dwellers aged 65 and above. In addition, participants in the present study were epigenetically younger compared to their chronological age, and very few of them were current or even former smokers. Smoking is one of the most detrimental lifestyle factors and is associated with an increased risk for several diseases (38), accelerated cellular aging (39), and mortality (40). Therefore, smoking behavior had to be acknowledged in the analyses. Additionally, it is noteworthy that smoking was taken into account in the development of DNAm GrimAge, the estimates of which represent combined information on chronological age, sex, and DNAm-based surrogate biomarkers for 7 plasma proteins and smoking pack-years (7). Previous studies have revealed that smoking behavior is a significantly stronger predictor of DNAm age than other lifestyle factors when GrimAge algorithm is used in estimation (9,41). Because our sample included relatively few smokers, the association of DNAm GrimAge age acceleration with mortality may be weaker than in populations with more smokers. The present study showed that 65% of the participants who were current smokers at the baseline level died during follow-up. However, due to the limited number of smokers (current smokers at baseline, *N* = 20), there was no statistically significant difference in the smoking status of the participants according to their vital status at the end of the follow-up period. Additionally, the participants in the present study were willing and able to travel to the laboratory and participate in tests of physical functioning. Thus, a selection bias of the participants could be considered another weakness of the study. On the other hand, it can be speculated that the present results are understated, and a more heterogeneous study group including more participants with unhealthy lifestyles, higher biological aging speed, and lower physical functioning could have strengthened the associations found in the present study.

Due to the limited size of the FITSA cohort, we were unable to conduct cause-specific mortality analyses, and thus only examined all-cause mortality. We conducted the analyses taking into account the effect of chronic diseases that were selected on the basis of their expected effect on epigenetic aging, physical functioning, and mortality. Further, we conducted sensitivity analyses among the participants with no reported chronic diseases, with the results supporting our main finding that both GrimAge and the TUG test were significant predictors of mortality. We consider the twin design as a major strength of the present study. Variations in both DNAm age (27,42) and physical function at older ages (25,26) are suggested to be influenced by genetic inheritance. We performed all individual-based analyses with adjustments for family relatedness. Being a MZ versus DZ twin was not associated with any outcomes in the individual analyses, indicating that twinship status is not affecting our individual-based results. In addition to individual-based analyses, we were able to conduct pairwise analyses. The power of this kind of within-pair analysis is that it controls for all unobserved factors constant within twin pairs (i.e., age, sex, cohort, and all genetic and shared environmental familial factors shared by the co-twins) (43). However, concern has been raised about the generalizability of twin findings to singleton populations due to the higher occurrence of, for example, reduced intrauterine growth and shorter gestation in twins compared to singletons (44). According to Barker’s hypothesis, low birth weight or low prenatal energy intake may lead to epigenetic modifications (45–47). Thus, one might think that the epigenetic profile and further morbidity and mortality of twins differ from that of singletons due to their prenatal conditions. However, large registry samples have shown that twins do not differ in morbidity or mortality from the rest of the population (48,49), supporting the generalizability of findings from twin studies. However, in the future, the association of the difference in birth weight and epigenetic aging of co-twins should be studied in more detail. Finally, a strength of the present study is the length of the follow-up period.

## Conclusion

This study supports earlier findings showing that accelerated epigenetic aging and lower physical functioning are associated with a higher risk of all-cause mortality. Further, the present findings suggest that DNAm GrimAge and the TUG test are strong predictors of mortality independent of each other and genetic influences among female twin pairs. DNAm-based measures and functional tests capture different aspects of the aging process and thus complement each other as measures of biological aging in predicting mortality. Further study is needed to determine how different physiological mechanisms interact to accelerate or delay aging.

## Supplementary Material

glad026_suppl_Supplementary_Material

## Data Availability

The mortality data used in the present study were obtained from Statistics Finland. The twin data set used in the current study will be located in the Biobank of the Finnish Institute for Health and Welfare, Finland. All the biobanked data are publicly available for use by qualified researchers following a standardized application procedure (https://thl.fi/en/web/thl-biobank/for-researchers).
