# Supplementary material for: Mortality Associations With DNA Methylation-Based Biological Aging and Physical Functioning Measures Across a 20-Year Follow-up Period
Source: J Gerontol A Biol Sci Med Sci. 2023 Jan 22;78(8):1489–96. doi: 10.1093/gerona/glad026 (PMC10395559; doi:10.1093/gerona/glad026)
Supplement: glad026_suppl_Supplementary_Material [file glad026_suppl_supplementary_material.pdf]

Online-Only Supplemental material for: Mortality associations with DNA methylation-based biological aging and physical functioning measures across a 20-year follow-up period

eTable 1. Risks of all-cause mortality per one standard deviation increase in DNAm GrimAge age acceleration, DunedinPACE, Timed Up and Go test, and 10-m walk test among female participants from the Finnish Twin Study on Aging (N = 395, age range 63–76 years) over a 20-year follow-up period……………………………………………………………………………………………...2

eTable 2. Risks of all-cause mortality per one standard deviation increase in DNAm GrimAge age acceleration, DunedinPACE, Timed Up and Go test, and 10-m walk test according to the tertiles of the predictors. The follow-up period for the female participants from the Finnish Twin Study on Aging (N = 395, age range 63–76 years) was from 2000 to 2020……………………………………………………………4

eTable 3. Biological aging and physical functioning (mean and standard deviation) data of the female participants from the Finnish Twin Study on Aging (N = 395, age range 63–76 years) according to the tertiles of DNAm GrimAge age acceleration, DunedinPACE, Timed Up and Go test, and 10-m walk test. Characteristics of the participants are presented overall and by vital status over a 20-year follow-up period……………………………………………………………………………………………...6

eTable 1. Risks of all-cause mortality per one standard deviation increase in DNAm GrimAge age acceleration, DunedinPACE, Timed Up and Go test, and 10-m walk test among female participants from the Finnish Twin Study on Aging (N = 395, age range 63–76 years) over a 20-year follow-up period.

	**Individual analyses**	**Pairwise analyses among twins**		
	(N = 395)	All (N = 186) twin pairs	Monozygotic (N = 91) twin pairs	Dizygotic (N = 95) twin pairs
**AAGrimAge**				
Model 1[a]	**1.36 (1.18-1.57)**	**1.92 (1.30-2.85)**	**2.08 (1.09-3.99)**	**1.83 (1.12-3.00)**
Model 1 + education	**1.33 (1.14-1.56)**	**2.00 (1.31-3.03)**	**2.09 (1.07-4.05)**	**1.95 (1.13-3.36)**
Model 1 + smoking	**1.39 (1.14-1.70)**	**1.74 (1.11-2.75)**	1.82 (0.86-3.88)	1.71 (0.93-3.14)
Model 1 + BMI	**1.36 (1.18-1.57)**	**1.98 (1.32-2.97)**	**2.66 (1.25-5.69)**	**1.83 (1.12-2.99)**
Model 1 + physical activity	**1.35 (1.17-1.56)**	**2.14 (1.40-3.28)**	**3.10 (1.39-6.90)**	**2.06 (1.19-3.57)**
Model 1 + lifestyle factors[b]	**1.44 (1.17-1.78)**	**2.24 (1.31-3.84)**	**4.11 (1.30-13.00)**	**2.25 (1.06-4.74)**
Model 2[c]	**1.39 (1.12-1.71)**	**2.18 (1.23-3.87)**	**5.78 (1.47-22.71)**	2.21 (0.96-5.10)
Model 2 + chronic diseases	**1.39 (1.11-1.74)**	**2.29 (1.23-4.25)**	**7.08 (1.60-31.19)**	2.05 (0.80-5.28)
**DunedinPACE**				
Model 1[a]	**1.23 (1.05-1.44)**	1.26 (0.84-1.88)	1.01 (0.51-1.98)	1.42 (0.86-2.35)
Model 1 + education	**1.18 (1.00-1.38)**	1.25 (0.83-1.89)	1.07 (0.54-2.13)	1.37 (0.81-2.31)
Model 1 + smoking	1.17 (0.99-1.38)	1.19 (0.79-1.81)	0.89 (0.43-1.83)	1.45 (0.84-2.49)
Model 1 + BMI	**1.25 (1.07-1.47)**	1.30 (0.86-1.96)	1.05 (0.53-2.10)	1.42 (0.84-2.40)
Model 1 + physical activity	**1.24 (1.05-1.46)**	1.27 (0.84-1.94)	1.20 (0.58-2.48)	1.53 (0.86-2.72)
Model 1 + lifestyle factors[b]	**1.22 (1.03-1.45)**	1.22 (0.78-1.92)	1.07 (0.49-2.34)	1.45 (0.77-2.73)
Model 2[c]	1.18 (0.99-1.40)	1.20 (0.75-1.92)	1.12 (0.50-2.50)	1.35 (0.69-2.62)
Model 2 + chronic diseases	1.15 (0.96-1.37)	1.14 (0.69-1.88)	1.19 (0.48-2.94)	1.13 (0.51-2.49)
**Timed Up and Go**				
Model 1[a]	**1.45 (1.32-1.59)**	**2.14 (1.40-3.27)**	**2.37 (1.19-4.72)**	**2.00 (1.17-3.43)**
Model 1 + education	**1.42 (1.30-1.56)**	**2.02 (1.30-3.13)**	**2.35 (1.17-4.71)**	**1.79 (1.00-3.19)**
Model 1 + smoking	**1.45 (1.32-1.59)**	**2.10 (1.36-3.23)**	**2.56 (1.21-5.40)**	**2.06 (1.78-3.61)**
Model 1 + BMI	**1.49 (1.35-1.65)**	**2.15 (1.41-3.27)**	**2.83 (1.27-6.33)**	**2.02 (1.17-3.48)**
Model 1 + physical activity	**1.46 (1.33-1.60)**	**2.25 (1.44-3.51)**	**3.48 (1.44-8.37)**	**1.84 (1.04-3.27)**
Model 1 + lifestyle factors[b]	**1.50 (1.36-1.65)**	**2.21 (1.39-3.50)**	**4.66 (1.61-13.49)**	**2.03 (1.06-3.90)**
Model 2[c]	**1.49 (1.35-1.64)**	**2.18 (1.34-3.57)**	**5.23 (1.70-16.09)**	**2.08 (1.01-4.28)**
Model 2 + chronic diseases	**1.43 (1.27-1.60)**	**2.34 (1.38-3.97)**	**5.77 (1.73-19.20)**	**2.82 (1.06-7.51)**

	Individual analyses (N = 395)	Pairwise analyses among twins		
		All (N = 186) twin pairs	Monozygotic (N = 91) twin pairs	Dizygotic (N = 95) twin pairs
**10-m walk**				
Model 1[a]	**1.45 (1.31-1.59)**	**2.27 (1.38-3.74)**	2.01 (0.99-4.07)	**2.52 (1.26-5.03)**
Model 1 + education	**1.43 (1.30-1.58)**	**2.56 (1.46-4.50)**	**2.05 (1.01-4.18)**	**3.41 (1.43-8.13)**
Model 1 + smoking	**1.46 (1.32-1.61)**	**2.12 (1.28-3.51)**	1.99 (0.96-4.09)	**2.20 (1.13-4.31)**
Model 1 + BMI	**1.52 (1.39-1.67)**	**2.38 (1.42-3.98)**	**2.50 (1.10-5.68)**	**2.52 (1.26-5.06)**
Model 1 + physical activity	**1.43 (1.28-1.60)**	**2.23 (1.33-3.74)**	**2.35 (1.02-5.40)**	**2.28 (1.13-4.63)**
Model 1 + lifestyle factors[b]	**1.51 (1.35-1.69)**	**2.11 (1.24-3.59)**	**2.95 (1.04-8.42)**	1.93 (0.95-3.91)
Model 2[c]	**1.51 (1.35-1.69)**	**2.62 (1.40-4.91)**	**2.86 (1.02-8.01)**	**2.84 (1.12-7.22)**
Model 2 + chronic diseases	**1.44 (1.26-1.63)**	**2.57 (1.35-4.89)**	**2.99 (1.03-8.64)**	2.84 (0.96-8.41)

Notes: AAGrimAge; GrimAge age acceleration; BMI, body mass index; DNAm, DNA methylation. [a]adjusted for family relatedness and age [b] adjusted for family relatedness, age, smoking (smoking status and pack-years), BMI, physical activity and alcohol consumption [c] adjusted for family relatedness, age, education, smoking, BMI, physical activity and alcohol consumption. Hazard ratios and 95% confidence intervals are presented in the table. Statistically significant values are bolded.

eTable 2. Risks of all-cause mortality per one standard deviation increase in DNAm GrimAge age acceleration, DunedinPACE, Timed Up and Go test, and 10-m walk test according to the tertiles of the predictors. The follow-up period for the female participants from the Finnish Twin Study on Aging (N = 395, age range 63–76 years) was from 2000 to 2020.

	AAGrimAge	DunedinPACE		TUG	10-m walk
**Model 1[a]**					
1 (slowest aging speed)	1	1	*1 (highest physical functioning)*	1	1
2 (medium aging speed)	1.17 (0.80-1.70)	1.19 (0.79-1.79)	*2 (medium physical functioning)*	1.34 (0.88-2.04)	1.20 (0.80-1.80)
3 (highest aging speed)	**1.68 (1.15-2.46)**	1.29 (0.87-1.91)	*3 (lowest physical functioning)*	**2.48 (1.71-3.59)**	**1.62 (1.10-2.40)**
**Model 1 + education**					
1 (slowest aging speed)	1	1	*1 (highest physical functioning)*	1	1
2 (medium aging speed)	1.09 (0.74-1.59)	1.07 (0.71-1.62)	*2 (medium physical functioning)*	1.35 (0.89-2.05)	1.12 (0.73-1.72)
3 (highest aging speed)	**1.55 (1.06-2.27)**	1.16 (0.78-1.73)	*3 (lowest physical functioning)*	**2.28 (1.57-3.31)**	**1.52 (1.00-2.31)**
**Model 1 + smoking**					
1 (slowest aging speed)	1	1	*1 (highest physical functioning)*	1	1
2 (medium aging speed)	1.14 (0.78-1.66)	1.15 (0.76-1.72)	*2 (medium physical functioning)*	1.31 (0.86-2.00)	1.25 (0.82-1.89)
3 (highest aging speed)	**1.52 (1.02-2.25)**	1.13 (0.76-1.69)	*3 (lowest physical functioning)*	**2.44 (1.67-3.56)**	**1.63 (1.08-2.46)**
**Model 1 + BMI**					
1 (slowest aging speed)	1	1	*1 (highest physical functioning)*	1	1
2 (medium aging speed)	1.18 (0.81-1.73)	1.21 (0.81-1.82)	*2 (medium physical functioning)*	1.44 (0.93-2.22)	1.24 (0.82-1.87)
3 (highest aging speed)	**1.72 (1.17-2.52)**	1.35 (0.91-1.99)	*3 (lowest physical functioning)*	**2.71 (1.86-3.98)**	**1.90 (1.26-2.86)**
**Model 1 + physical activity**					
1 (slowest aging speed)	1	1	*1 (highest physical functioning)*	1	1
2 (medium aging speed)	1.17 (0.81-1.68)	1.20 (0.79-1.81)	*2 (medium physical functioning)*	1.34 (0.87-2.07)	1.17 (0.78-1.76)
3 (highest aging speed)	**1.72 (1.17-2.51)**	1.27 (0.85-1.89)	*3 (lowest physical functioning)*	**2.44 (1.66-3.58)**	**1.52 (1.00-2.32)**
**Model 1 + lifestyle factors[b]**					
1 (slowest aging speed)	1	1	*1 (highest physical functioning)*	1	1
2 (medium aging speed)	1.13 (0.78-1.64)	1.19 (0.79-1.80)	*2 (medium physical functioning)*	1.37 (0.88-2.15)	1.23 (0.79-1.92)
3 (highest aging speed)	**1.60 (1.06-2.40)**	1.18 (0.78-1.77)	*3 (lowest physical functioning)*	**2.59 (1.73-3.89)**	**1.75 (1.08-2.82)**

	AAGrimAge	DunedinPACE		TUG	10-m walk
**Model 2[c]**					
1 (slowest aging speed)	1	1	*1 (highest physical functioning)*	1	1
2 (medium aging speed)	1.08 (0.74-1.57)	1.10 (0.73-1.66)	*2 (medium physical functioning)*	1.40 (0.90-2.18)	1.17 (0.74-1.84)
3 (highest aging speed)	**1.51 (1.00-2.26)**	1.09 (0.71-1.66)	*3 (lowest physical functioning)*	**2.46 (1.65-3.66)**	**1.68 (1.03-2.75)**
**Model 2 + chronic diseases**					
1 (slowest aging speed)	1	1	*1 (highest physical functioning)*	1	1
2 (medium aging speed)	1.09 (0.76-1.58)	1.06 (0.70-1.62)	*2 (medium physical functioning)*	1.49 (0.95-2.34)	1.09 (0.69-1.74)
3 (highest aging speed)	1.44 (0.95-2.17)	0.99 (0.64-1.54)	*3 (lowest physical functioning)*	**2.26 (1.50-3.41)**	1.41 (0.85-2.35)

Notes: AAGrimAge, GrimAge age acceleration; BMI, body mass index; DNAm, DNA methylation. [a]adjusted for family relatedness and age [b] adjusted for family relatedness, age, smoking (smoking status and pack-years), BMI, physical activity and alcohol consumption [c] adjusted for family relatedness, age, education, smoking, BMI, physical activity and alcohol consumption. Hazard ratios and 95% confidence intervals from the individual-based analyses are presented in the table. Statistically significant values are bolded.

eTable 3. Biological aging and physical functioning (mean and standard deviation) data of the female participants from the Finnish Twin Study on Aging (N = 395, age range 63–76 years) according to the tertiles of DNAm GrimAge age acceleration, DunedinPACE, Timed Up and Go test, and 10-m walk test. Characteristics of the participants are presented overall and by vital status over a 20-year follow-up period.

**Characteristic**	**All** (N = 395)	**Alive at the end of the follow-up** (N = 208)	N (%)	**Dead** (N = 187)	N (%)
**AAGrimAge**					
1 (slowest aging speed)	-3.11 (1.14)	-3.2 (1.2)	82 (62.1)	-3.0 (1.1)	50 (37.9)
2 (medium aging speed)	-0.52 (0.61)	-0.5 (0.6)	69 (52.7)	-0.6 (0.6)	62 (47.3)
3 (highest aging speed)	3.35 (2.73)	2.8 (2.5)	57 (43.2)	3.7 (2.9)	75 (56.8)
**DunedinPACE**					
1 (slowest aging speed)	0.86 (0.05)	0.85 (0.05)	83 (62.9)	0.86 (0.04)	49 (37.1)
2 (medium aging speed)	0.97 (0.03)	0.97 (0.03)	65 (49.6)	0.97 (0.03)	66 (50.4)
3 (highest aging speed)	1.09 (0.06)	1.08 (0.05)	60 (45.5)	1.1 (0.06)	72 (54.5)
**Timed Up and Go** (seconds)					
1 (highest physical functioning)	7.5 (0.6)	7.4 (0.6)	89 (67.4)	7.7 (0.5)	43 (32.6)
2 (medium physical functioning)	8.9 (0.4)	8.9 (0.3)	73 (55.3)	9.0 (0.4)	58 (44.7)
3 (lowest physical functioning)	11.3 (1.8)	10.9 (0.8)	46 (34.8)	11.6 (2.1)	86 (65.2)
**10-m walk** (seconds)					
1 (highest physical functioning)	4.8 (0.4)	4.7 (0.4)	85 (64.4)	5.0 (0.3)	47 (35.6)
2 (medium physical functioning)	5.8 (0.3)	5.8 (0.3)	70 (53.4)	5.9 (0.3)	61 (46.6)
3 (lowest physical functioning)	7.4 (1.2)	7.0 (0.6)	53 (40.2)	7.7 (1.4)	79 (59.8)

Notes: AAGrimAge, GrimAge age acceleration; DNAm, DNA methylation. Values are means and standard deviations.
